# Supplementary material for: Decoding the Fundamental Drivers of Phylodynamic Inference
Source: Mol Biol Evol. 2023 Jun 2;40(6):msad132. doi: 10.1093/molbev/msad132 (PMC10284498; doi:10.1093/molbev/msad132)
Supplement: msad132_Supplementary_Data [file msad132_supplementary_data.pdf]

May 29, 2023

Supplementary Figures

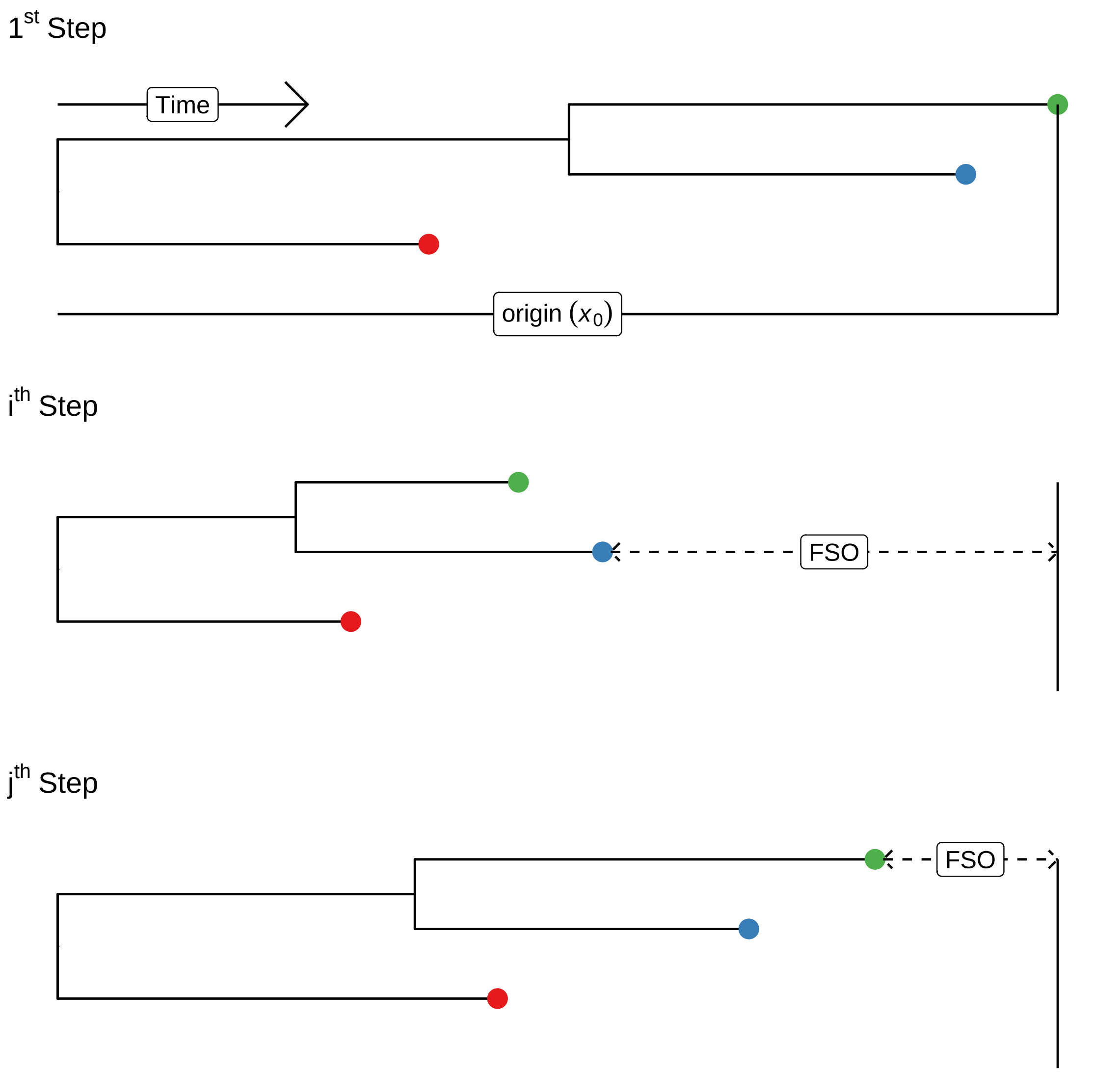

Figure S1: Visual intuition for the novel tip-date operator that allows for inference from sequence only datasets. Each tree represents a state/step in an hypothetical MCMC chain. The Final Sample Offset (FSO) is the time between the final sample date and the end of the fitted birth-death process. It is normally held constant in inference as a nuisance parameter. The tip date operator instead allows for new proposals of the FSO. This allows the total time taken for the fitted process to vary. Tip dates are also varied relative to each other, such that any tip can take the final sampling time. Overall, tip dates can vary in order, but also in absolute time due to the FSO. The changing order of coloured tips and changing timespan of each tree/step is intended to reflect this. The origin time is still measured as the time from the root to the time of the final sample. The FSO and tip dates are therefore additional parameters for which proposals are generated.

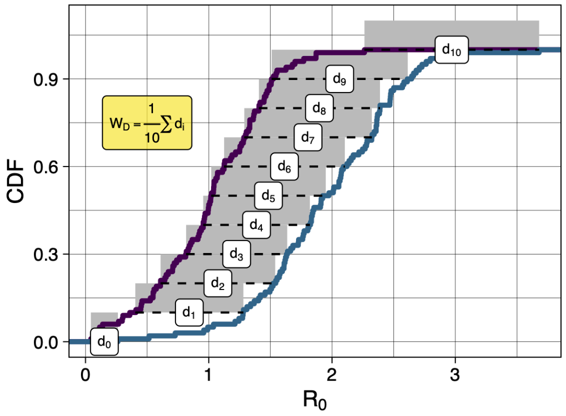

Figure S2: Visual intuition for calculation of the Wasserstein metric inspired by Kolouri et al. (2019). It can be thought of as integration over the horizontal distance between points of the cumulative distribution function. For two sets of posterior samples from an MCMC, the 10 integration bins would be replaced by the the size of the greatest set of samples.

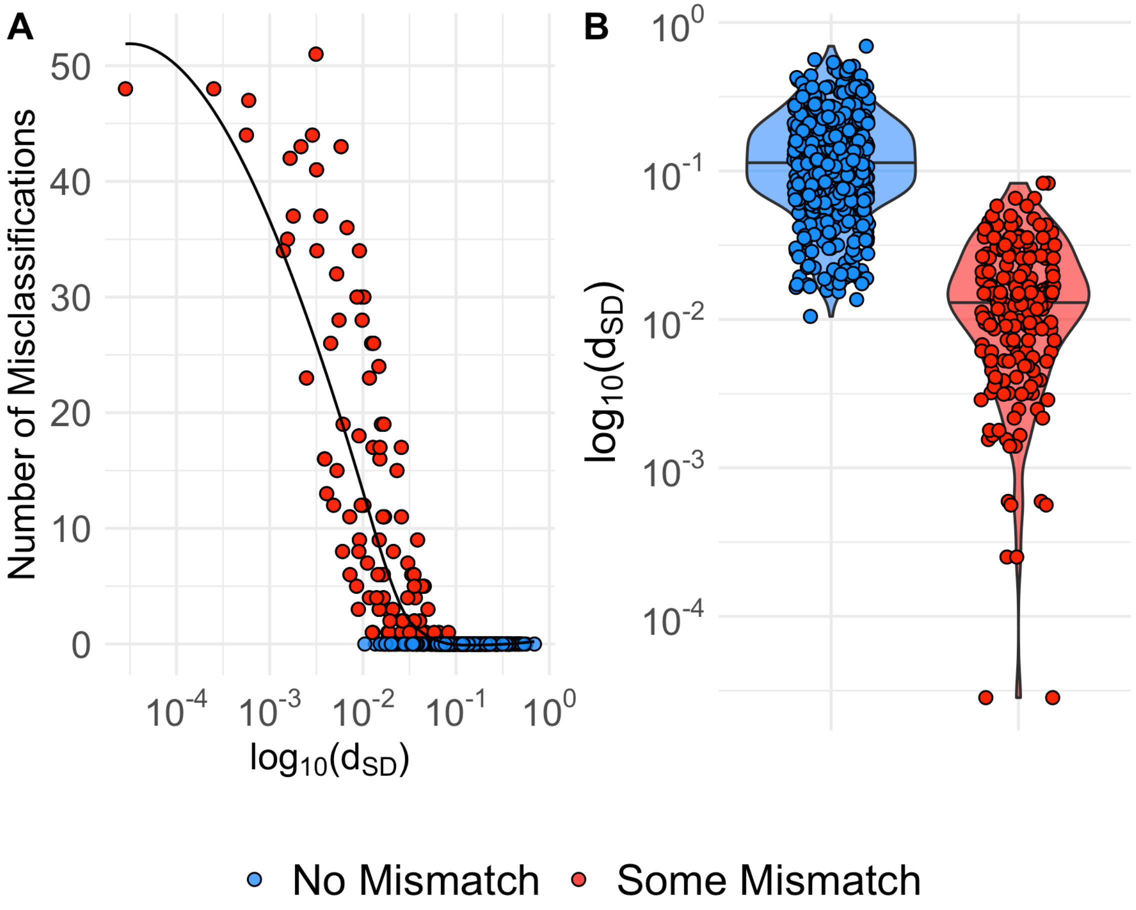

Figure S3: **A**) Each point presents the number of misclassification in subsampling posterior R_0 for each of the 600 simulated datasets. X-axis is the log transformed difference between W_S and W_D. **B**) Violin plots with jittered points of the difference between W_S and W_D for simulated alignments where there was wither some one no misclassification. Both **A** and **B** support that misclassication only occurs where the difference between W_S and W_D is negligible, and classification is not meaningful in the first instance.

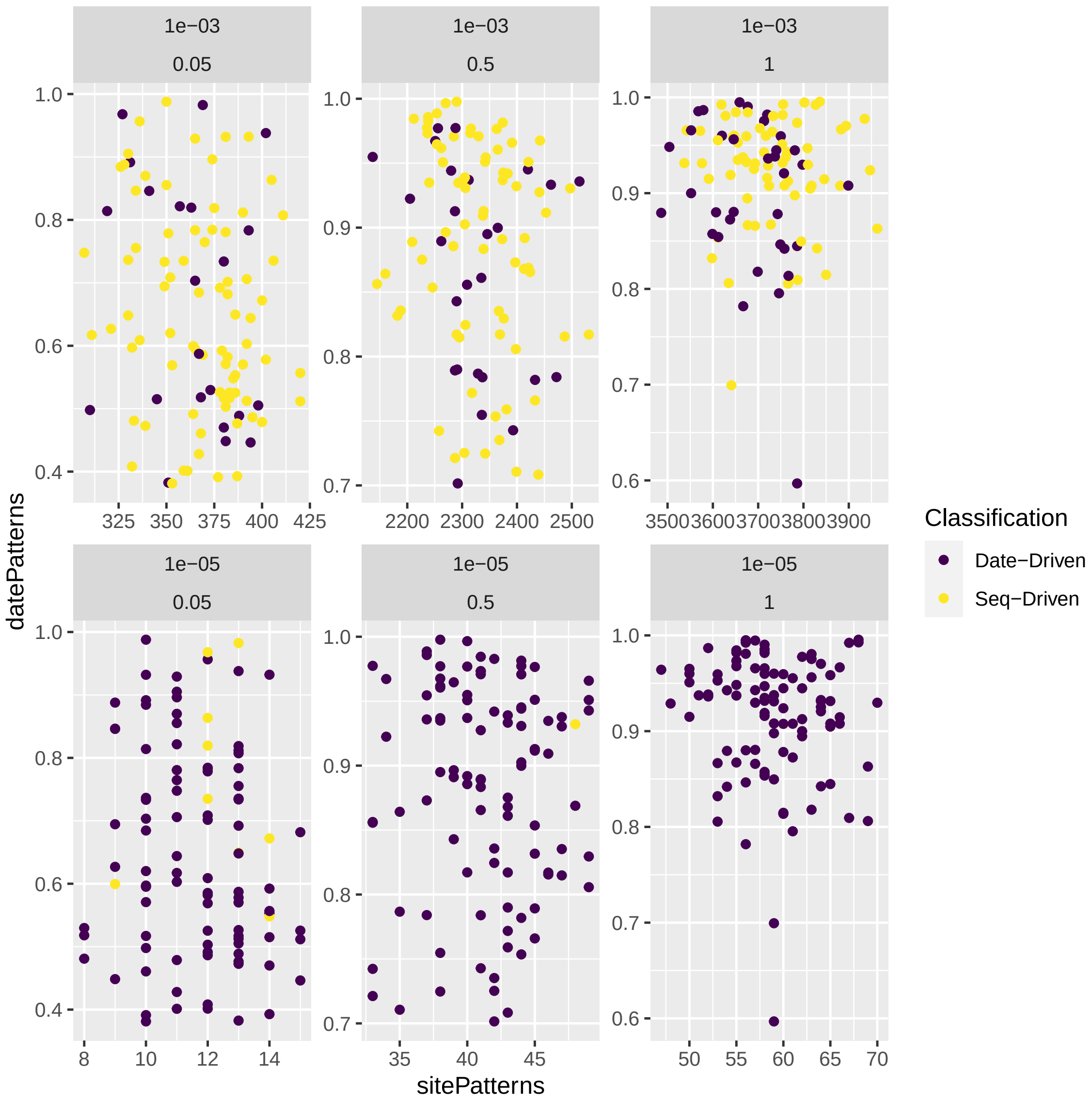

Figure S4: Date patterns ($= \frac{\text{Sampling Span}}{\text{Height}}$) against site patterns for each simulated dataset. Plots are separated by evolutionary rate and sampling proportion such that there are 600 points coloured by Wasserstein Classification across the entire figure. Higher evolutionary rates increase the proportion of Sequence driven datasets, but within each rate there is no clear pattern in date patterns, site patterns, or sampling rate driving classification.

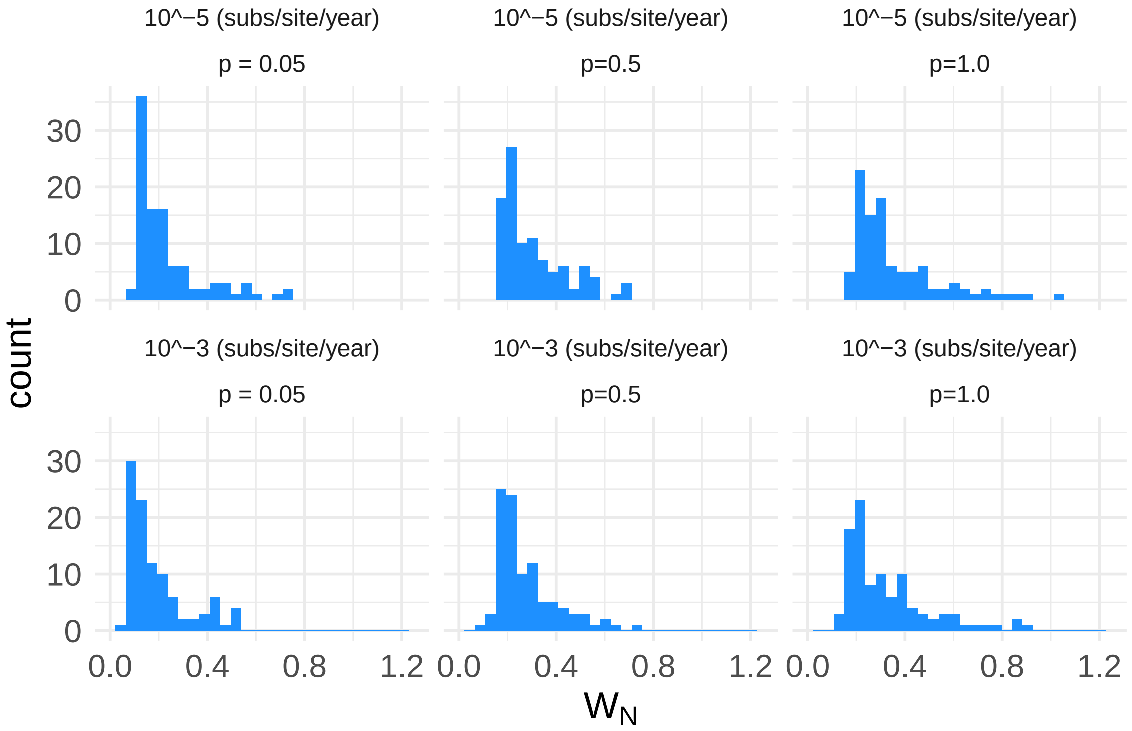

Figure S5: Histogram of W_N for each simulated dataset, separated by evolutionary rate and sampling proportion. W_N ranges from 0.06 to 1.02, such that simulated data provide additional information beyond that of the prior in each analysis.

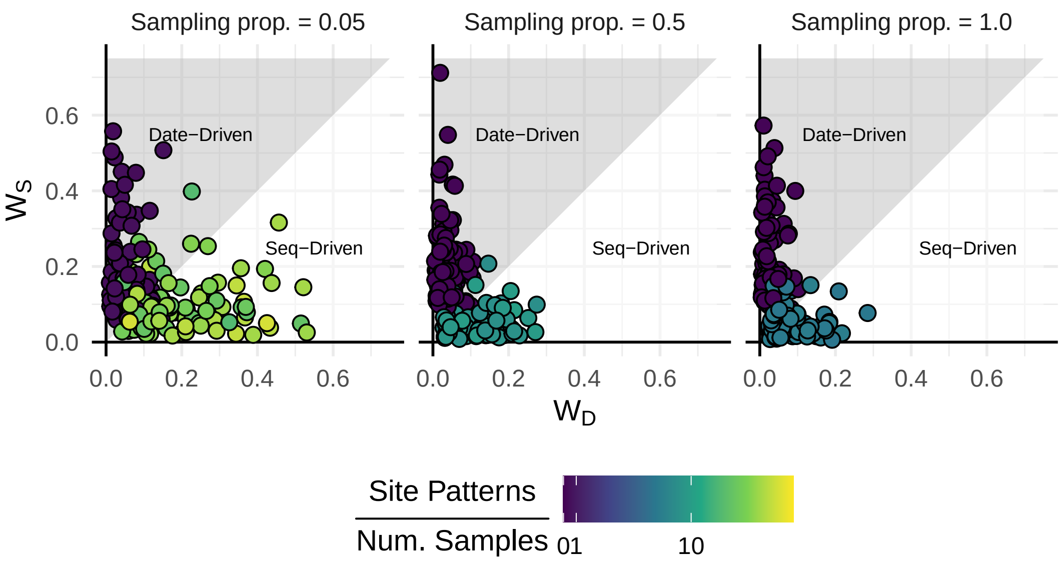

Figure S6: Number of site patterns per sample across each sampling proportion used in the simulation study. Analyses classified as sequence driven always have more than one site pattern per sample. This is not a sufficient condition to an analysis being sequence driven, as seen in some analyses with higher site patterns per sample still being classified as date driven, but appears as a necessary condition.

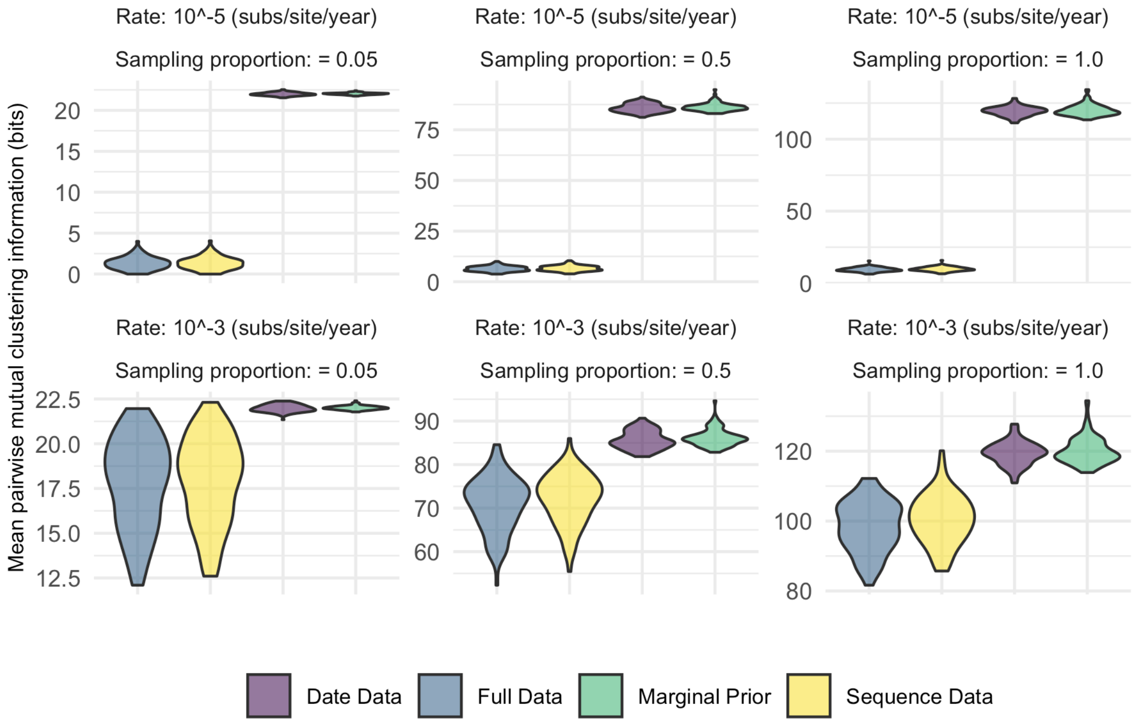

Figure S7: Violin plots of pairwise mutual clustering information tree difference for 100 trees sampled posterior tree distributions for each simulated dataset. 100 trees were samples from each of the 600 posterior tree distributions a total of 100 times, resulting in 60,000 datapoints here, separated by the sampling proportion and evolutionary rate used in each simulation. Uniformly, Full data has the least tree-distance between posterior trees followed by the sequence-only, dates-only, and no-data treatments.

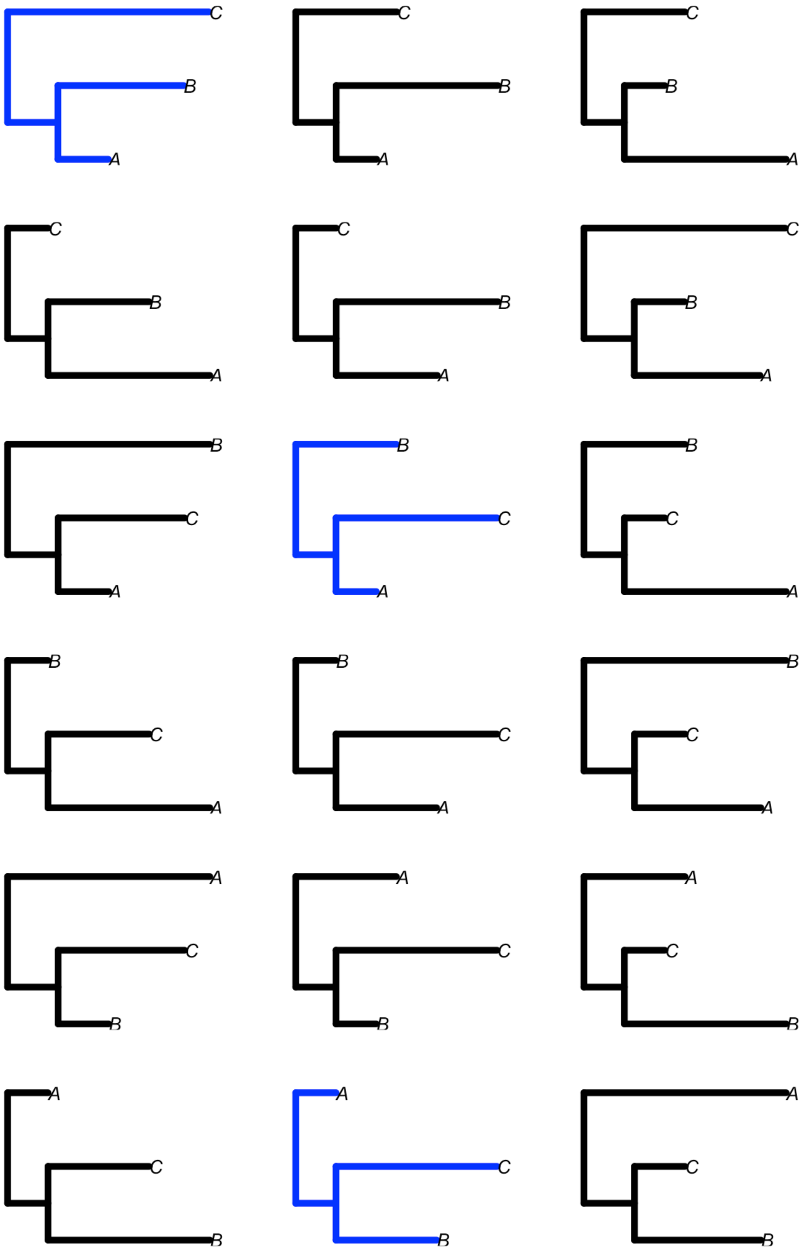

Figure S8: A representation of ranked tree space for a dataset of 3 taxa. Branch lengths are in units of time. There are a total of 18 possible ranked topologies, which would be the size of tree space for an analyses of a sequence-only dataset with three samples. Trees in blue are those with the sampling chronology A, B, and then C. These represent the tree space for a hypothetical data-only dataset with three known sampling times. The key message is that tree space for sequence-only data is much larger than for date data, even with as little as three tips. Moreover, the disparity between the sequence-only tree space and date-only space grows with sample size, such that the state-space for sequence-only data is orders of magnitude larger than that for dates-only at the size of a regular phylodynamics dataset.

References

S. Kolouri, K. Nadjahi, U. Simsekli, R. Badeau, and G. K. Rohde. Generalized Sliced Wasserstein Distances, Feb. 2019. URL http://arxiv.org/abs/1902.00434. arXiv:1902.00434 [cs, stat].
